# Cultural values, national personality characteristics, and intelligence as correlates of corruption: A nation level analysis

**DOI:** 10.1016/j.heliyon.2022.e09506

**Published:** 2022-05-21

**Authors:** Esma Gaygısız, Timo Lajunen

**Affiliations:** a Department of Economics, Middle East Technical University, 06531, Ankara, Turkey; b Department of Psychology, Norwegian University of Science and Technology, 7491, Trondheim, Norway

**Keywords:** Corruption, Culture, Personality, Gross domestic product, IQ

## Abstract

Research on national corruption has primarily focused on economics and politics, whereas cultural factors and especially national personality characteristics have attracted less attention. In the present study, the influence of Hofstede's cultural dimensions, Schwartz's values, Eysenckian personality factors (EPQ), and national intelligent quotient (IQ) on Corruption Perception Index (CPI) scores were studied by using aggregated data from 64 countries and ecological design, i.e., correlations, partial correlations, multiple regression analyses, and mediation and moderation analyses. The sub-datasets included 35 countries for the EPQ, 46 countries for the Hofstede cultural values, 30 countries for the Schwartz value dimensions, and all 64 countries for the IQ. Both partial correlations and regression analyses in which GDP per capita was controlled emphasized the importance of national income in corruption so that high income was negatively related to CPI scores. Regression analysis results showed that the EPQ Lie scores, Hofstede's power distance, and masculinity, as well as Schwartz's hierarchy, mastery, and harmony, were positively, and individualism and intelligence negatively related to corruption. The mediation analyses showed that the effects of cultural values on corruption were mostly mediated by income (GDP per capita). Hence, the national culture influences the national income level, which, in turn, is related to corruption. The effect of individualism on corruption was moderated by income so that individualism was negatively related to corruption in countries with lower income. The results suggest that cultural factors should be considered in corruption studies and anti-corruption policies. Without taking into account the national cultural values, anti-corruption policies might not be perceived well by the public, compromising the effects of the interventions.

## 1 Introduction

Economists and social scientists generally see that corruption has adverse effects on economic growth as well as on political and social development (Johnston, 1998; Shen, 2007). Corruption has adverse effects in many political and economic aspects, including the effectiveness of government (Berkovich, 2016), economic growth (Haruna and Abu Bakar, 2021; Song et al., 2021), foreign investments (Ledyaeva et al., 2013; Zhu and Shi, 2019), tax compliance and revenue (Bertinelli et al., 2020; Irawan and Utama, 2021), quality of infrastructure (Bertinelli et al., 2020), and public services including education and health care (Nguyen et al., 2017; Setyaningrum et al., 2017), and effectiveness of aid-funded projects (Shen, 2007). In addition to these direct effects on an economy and a political system, corruption reduces citizens' trust in their government's ability to provide equal services for all citizens (Khan et al., 2021; Silal and Saha, 2021).

One of the most widely used definitions for corruption is that “corruption refers to acts in which the power of the public office is used for personal gain in a manner that contravenes the rules of the game" (Jain, 2001). Hence, according to this definition, corruption always involves the misuse of public power for personal gains. Corruption Perceptions Index (CPI), published by Transparency International (Transparency International, 2020), provides a reasonable general estimate of corruption at a macro level in a country.

One of the most extensive studies about economic and non-economic correlates of corruption included 193 observations and 70 variables (Seldadyo and de Haan, 2006). According to the results, the most robust determinates of corruption were regulatory capacity, population density, Scandinavian legal origin, ethnic tension, socialism legal origin, the proportion of the population with no religion, ethnic conflict, illiteracy rate, government wage, latitude, fuel export, primary school enrolment, external debt, and proportion of women in the labour force (Seldadyo and de Haan, 2006). In another study about the causes of corruption, Protestant traditions and histories of British rule were related to low corruption (Treisman, 2000). In Paldam's study (Paldam, 2001), religion had an additional effect of 9% in corruption score after the effects of economic conditions (71% of variance accounted for) had been taken into account. Based on these few studies, it can be assumed that cultural variables and national character contribute to the corruption level in a country. Cultural factors may form the "distal factors," which influence corruption partly via "proximal factors" like economic policies. Political and economic decisions should reflect the public's opinion to some degree.

### 1.1 Hofstede's cultural dimensions and corruption

Geert Hofstede (2001) called culture “the collective programming of the mind that distinguishes the members of one group or category of people from another." The culture consists of “a system of societal norms consisting of the value systems (or the mental software) shared by major groups in the population” (G. Hofstede, 2001). The five Hofstede's value dimensions are presented in Table 1.

**Table 1:** Study variables.

Variable	Content
Corruption Perception Index (CPI)	The perceived levels of public sector corruption
Gross Domestic Product per capita (GDP)	A country's economic output per person
**Hofstede's values**	
Power distance (PDI)	The amount to which the less powerful members of groups and cultural units accept that power is distributed unevenly
Individualism (IDV)	The extent to which people are seen as self-reliant independent agents who choose their personal affiliations
Masculinity (MAS)	The value placed on traditionally “male” values: competitiveness, assertiveness, ambition, and having wealth and material possessions
Uncertainty avoidance (UAI)	The extent to which people attempt to cope with anxiety by reducing uncertainty; preference of rules and structured settings
**Schwartz values**	
Harmony	Discouraging efforts to generate change and encouraging conservation of smooth relations
Embeddedness	Seeing people as part of the collective; preserving the status quo and traditional order
Hierarchy	Reliance on hierarchy of given roles; seeing the unequal distribution of power, roles, and resources as rightful
Mastery	Active self-assertion to master, direct, and change the natural and social environment to achieve personal aims
Affective autonomy	Encouraging people to pursue exciting, affectively positive personal experiences
Intellectual autonomy	Encouraging people to pursue their own ideas and intellectual directions autonomously
Egalitarianism	Recognizing another person as moral equal who shares the same basic interests as a human; comitement to cooperation with others; being concerned for the wellbeing of other people; volunteering for the common good
**EPQ**	
Psychoticism (P)	Tough-mindedness, nonconformity, inconsideration, irresponsibility, lack of sympathy, antagonism, and recklessness
Extraversion (E)	sociability, spontaneity, lack of shyness, vivacity, confidence, and inventiveness
Neuroticism (N)	anxiousness, irritability, tenseness, shyness, moodiness, lack of self-esteem
Lie (L)	Dissimulation tendencies
**IQ**	Intelligence quotient

Earlier studies about Hofstede's cultural dimensions and corruption have had somewhat mixed results. In some studies, high IDV has been negatively related to corruption (Connelly and Ones, 2008; Davis and Ruhe, 2003; Tu et al., 2020; Yeganeh, 2014), while in some other studies, no relationship between IDV and corruption has been reported (G. Hofstede, 2001; Husted, 1999). As Husted (1999) proposed, the lack of IDV and corruption relationships might be due to the high correlation between IDV and national income, which means that GDP as a stronger predictor supersedes the IDV. However, it is possible that IDV influences corruption via GDP, which would refer to the mediator role of the GDP. Results about UAI and PDI and corruption are much more homogeneous in the research literature. Several studies have reported positive relationships between PDI and corruption (Cheung and Chan, 2008; Connelly and Ones, 2008; Davis and Ruhe, 2003; Franke and Nadler, 2008; G. Hofstede, 2001; Husted, 1999; Yeganeh, 2014) and UAI and corruption (Connelly and Ones, 2008; Davis and Ruhe, 2003; Franke and Nadler, 2008; G. Hofstede, 2001; Husted, 1999; Yeganeh, 2014) although in one study UAI was not related to corruption (Cheung and Chan, 2008). Results about the last of Hofstede values, MAS, have been more mixed than those related to PDI and UAI. In some studies, MAS has been positively related to corruption (Davis and Ruhe, 2003; G. Hofstede, 2001; Tu et al., 2020; Yeganeh, 2014), whereas some other studies did not find any relationship between MAS and corruption (Cheung and Chan, 2008; Connelly and Ones, 2008; Franke and Nadler, 2008) or the correlation was weak (Davis and Ruhe, 2003). In conclusion, we could say that PDI and UAI are strongly related to corruption, but the relationship between MAS and corruption is still open. IDV seems to correlate strongly with corruption when GDP or Gross National Product (GNP) is not entered into the model. The relationships between IDV, national income, and corruption need to be studied further.

The strong relationship between corruption and PDI and UAI found in previous studies is based on the hierarchical and inflexible nature of a society scoring high in PDI and UAI. In this kind of culture, the basic idea is that inequality is fundamentally justified, the powerful are entitled to privileges, and that citizens are not equal in front of the law. In addition, questioning the status quo and prevailing practices is considered anxiety-provoking and threatening to society. In collectivist societies, in-group loyalty is highly valued, and the family and friends create a lasting network in which favours and services are exchanged. In addition to this mutual exchange, in-group cohesion supported by collectivist values imply that different standards are applied when evaluating the behaviour of different groups (Davis and Ruhe, 2003). It can be expected that the frequent mutual exchange of favours and relativity of standards in collectivistic societies makes corruption more common. In previous studies, masculinity (MAS) has had a complicated relationship with corruption. Masculine countries emphasize such values as competitiveness, assertiveness, materialism, and lack of concern for other people. Since material success, especially for men, is a highly valued characteristic in a masculine society, we can assume that corruption as a means to success is more common in masculine societies (Weaver, 2001).

### 1.2 Schwartz's cultural values and corruption

Schwartz's cultural values are based on three main challenges that all societies face. The first challenge is to decide in which degree people are autonomous individuals or embedded in their group. This dimension can be described by using value categories "conservatism,” “intellectual autonomy,” and “affective autonomy.” The second concern is to “preserve the social fabric” by guaranteeing responsible behavior (S. H. Schwartz, 1994). "Hierarchy" and "egalitarianism" are two main means to conserve the society's social structure. The third challenge concerns how individuals relate to their environment including both natural and social environment. This individual-environment relationship is based on values “mastery” and “harmony” (Schwartz, 1994). A detailed description of Schwartz's cultural values can be found in Table 1.

So far, Schwartz's value theory has been utilized much less in corruption research than Hofstede's model. In a study by Gouveia (2002), the relationships between Schwartz's value types and “institutional index,” i.e., a composite score of institutional development, were studied. Corruption was one of the four measures used to calculate the "institutional index.” Results showed that Egalitarian Commitment was positively related to higher institutional development (and lack of corruption as part of it), whereas Conservatism was negatively related to institutional index score (Gouveia, 2002). However, it should be noted that corruption was only one of the four scores used to calculate the index score and, therefore, a direct relationship between corruption and Schwartz's value scores was not studied. In his study, Yeganeh (2014) concluded that Schwartz's values autonomy and mastery “tend to impede corruption” (Yeganeh, 2014).

Two studies have addressed the relationship between Schwartz's cultural dimensions and the "culture of corruption" at the corporate level. Mazzi, Slack, and Tsalavoutas (Mazzi et al., 2018) used a panel dataset of 222 European firms to investigate the relationship between Schwartz's cultural dimensions and the levels of compliance with mandatory disclosure requirements by International Financial Reporting Standards. According to their results, hierarchy and mastery were associated with lower compliance levels. Agyei-Mensah and Buertey (Agyei-Mensah and Buertey, 2019) investigated the relationships between corruption, cultural values, and corporate social performance among 335 most active-traded companies listed on the stock exchanges of South Korea, Egypt, Kenya, Nigeria, and South Africa. Especially mastery (p = -0.78) and embeddedness (p = 0.69) correlated with corruption.

The first dimension in Schwartz's value theory (Conservatism vs autonomy) can be expected to be related to corruption so that cultures emphasizing conservatism score higher on corruption. For conservative societies, in-group solidarity is a more important value than fairness and equality. In other words, such values as "fairness" are evaluated differently for in-group than out-group members, which can lead to in-group favouritism and, thus, corruption for the benefit of in-group members. In the second dimension, "hierarchy vs egalitarianism," we can expect hierarchical societies to be more prone to corruption than egalitarian societies. Hierarchical societies rely on hierarchical systems with rigid roles for individuals to ensure responsible behaviour. The unequal distribution of power and resources is seen as legitimate and even necessary for the smooth functioning of society. Opposite to hierarchical societies, members of egalitarian societies accept individuals as moral equals who share fundamental interests as citizens. Since corruption is based on an unequal distribution of power and resources and, thus, violates common fairness and other people's rights, the hierarchy should have a positive and egalitarianism negative relation to corruption. The third societal dilemma in Schwartz's value theory is related to mastery vs harmony. A society characterized by mastery values emphasizes active self-promotion (by individuals or groups) and the right to change the environment to reach one's goals. On the other hand, cultures emphasizing harmony urge maintaining smooth conflict-free relations. Since mastery values bear the idea that an individual should pursue his or her goals assertively and often by any means, including illegal actions, mastery should be positively related to corruption.

### 1.3 National personality characteristics and corruption

The Eysenck Personality Questionnaire (Eysenck and Eysenck, 1975) was composed to measure E (extraversion vs introversion), N (neuroticism vs emotional stability), P (psychoticism vs ego control) and L (lying) (Table 1). Lynn and Martin (Lynn and Martin, 1995) studied the demographic and socio-economic factors related to E, N, and P in a set of 37 countries. According to their findings, extraversion was positively related to competitiveness and work ethics. Neuroticism was related to anxiety, alcoholism, and income per capita, while psychoticism correlated with competitiveness (Lynn and Martin, 1995). In their study using data from 34 countries, Barrett, Petrides, Eysenck, and Eysenck (Barrett et al., 1998) confirmed the cross-cultural validity of the EPQ factor structure, including four factors (E, N, P, and L).

Van Hemert and colleagues (van Hemert, van de Vijver, Poortinga and Georgas, 2002) used data from 24 countries to investigate correlations between the EPQ scale scores and a wide range of social and economic indicators. Corruption was measured with the Bribe Payers Index and CPI so that a high score indicated a high level of corruption. Results showed strong positive relationships between the P and L and Bribe Payers Index scores as well as the negative relationship between E and Bribe Payers Index. CPI correlated positively with L (van Hemert et al., 2002). In the study by Connelly and Ones (Connelly and Ones, 2008), corrupt countries seemed to score higher in conscientiousness and neuroticism and lower in openness. Extraversion was negatively related to corruption.

Eysenck's personality theory has two scales, psychoticism and lie, which can be expected to be related to corruption. Since P is composed of antisocial tendencies (e.g., nonconformity, irresponsibility, lack of respect for norms and rules), P is likely to be related to corruption. The second Eysenckian factor supposedly related to corruption is the Lie scale. The EPQ Lie scale is a scale measuring a personality characteristic of “trying to look better than actually is” in front of others. The Lie scale is considered to be more than merely a measure of dissimulation but rather reflects some stable personality dimension in its own right (Furnham et al., 2008). Corruption is deceptive behaviour in which the rules and the principle of fairness are violated. We can expect cultures in which rule obedience and honesty are not primary values to show a higher amount of corruption.

### 1.4 Intelligence

In their “IQ and the wealth of nations” (Lynn and Vanhanen, 2002) and “IQ & Global Inequality” (Lynn and Vanhanen, 2006), Richard Lynn and Tatu Vanhanen reported extremely high correlations (0.70 and 0.68) between intelligence and a nation's wealth underlining the importance of intelligence as one factor in accounting for disparities between rich and developing countries. Among several indicators of development and well-being, Lynn and Vanhanen (2002, 2006) also studied the association between IQ and corruption. Lynn and Vanhanen (2002, 2006) assumed a causal relationship between IQ and corruption so that “nations with high national IQs have better chances to root out corruption than the nations with low national IQs” (2006, p. 209). Hence, countries with high IQ should be better equipped in preventing corruption and constructing well-working organizations in which corruption is not needed. Indeed, IQ had a strong correlation with CPI, indicating that corruption tends to be more prevalent in countries with low national IQ than in countries with high national IQ.

### 1.5 The aim of the study

The first aim of this study was to investigate the relationships between cultural variables (Hofstede's and Schwartz's variables), national characteristics (Eysenck's personality factors and IQ), and corruption (CPI). Cultural theories (Hofstede, Schwartz, EPQ, IQ) naturally have overlap with each other. Therefore, the second aim was to compare these models to see which of them is the best predictor of corruption at the country level. None of the previous studies investigated the possibility that cultural dimensions and national characteristics could influence corruption via a mediator variable such as GDP per capita. In this model, culture would contribute to income level, which in turn would be related to corruption. Alternatively, the level of GDP of a country may moderate the relationship between culture and corruption. For example, a significant relationship between cultural variables and corruption could apply only in the case of low-income countries but not among high-income countries in which the official regulatory bodies have a stronger role in economic activities. A large body of studies has shown that national income (GDP) has a strong negative relationship to corruption level and that certain cultural factors (e.g., individualism) correlate with corruption. Therefore, the third aim was to investigate the mediator/moderator role of GDP in culture – corruption relationship. To our knowledge, this is the first study in which the major cultural models (Hofstede, Schwartz), national personality characteristics (EPQ), and national intelligence score (IQ) are compared in terms of corruption.

## 2 Method

The study applied an ecological design, i.e., correlation-based analyses. The selection of cases (i.e., countries) was based on a simple criterion that all countries having data were included in the analysis. No sub-sampling or restrictions were applied, so the availability of the data determined the final sample size. The scores were used as reported in the original sources. To guarantee the methodological homogeneity and, thus, reliability of the scores, we did not add new studies but used the values reported in the original studies by Hofstede (2001), Schwartz (1994), van Hemert & al. (2002), Lynn and Vanhanen (2006), which provided mean country scores.

The country-level indicators were obtained for 64 countries from which 35 countries were used for the EPQ analyses, 46 countries for the analyses involving Hofstede's dimensions, 30 countries for the Schwartz's values, and all 64 countries for the IQ. Variables included in the study were Extraversion (E), Psychoticism (P), and Neuroticism (N) and Lie (L) from the EPQ; Power Distance (PDI), Uncertainty Avoidance (UAI), Individualism (IDV), Masculinity and (MAS) from Hofstede's Culture Questionnaire; and Conservatism, Intellectual Autonomy, Affective Autonomy, Hierarchy, Egalitarianism, Mastery, and Harmony from Schwartz Value Questionnaire, and IQ from Lynn's and Vanhanen's (2006) study. In addition, GDP per capita and the Corruption Perception Index (CPI) were obtained for each country. In the original CPI, a high CPI score actually means low corruption. Since this complicates the interpretation of the results, the direction of the CPI scores was changed so that a high CPI score indicated a high level of corruption (van Hemert et al., 2002).

The national EPQ N, E, P and L scores were obtained from the study of van Hemert & al. (2002). Van Hemert & al. reviewed all studies published about the EPQ, which provided data for 333 separate samples from 38 countries with a total of 68,374 respondents (45.6% male; mean age 27.46 years and standard deviation 9.30 years). Scores for Hofstede's culture dimensions were obtained from Hofstede (Hofstede, 1980; Hofstede, 2001). Data collection methods and the “functional equivalence of matching samples” (Hofstede, 2001, p. 23) are explained in detail in Hofstede & Minkov (G. Hofstede and Minkov, 2013). The Schwartz value scores were downloaded from (S. H. Schwartz, 1994). Similarly, as with the Hofstede measures, Schwartz secures the comparability of samples collected from different countries by matching the samples carefully. A more detailed description of the development of the instrument, data collection methods and the samples can be found in (Shalom H. Schwartz et al., 2012) and in (S. H. Schwartz, 1994). IQ values were obtained from (Lynn and Vanhanen, 2006). The IQ scores were based on measurements done by standardized IQ tests in 113 nations (Lynn and Vanhanen, 2006). GDP per capita figures for 2002–2004 were obtained from the United Nations Statistics Division and the Corruption Perception Index for 2002–2004 from Transparency International (Transparency International, 2002, 2003, 2004). Statistics were obtained for a period of three years, and an average was calculated. These years were selected because they were close to the years when cultural values, personality factor scores and IQ scores were measured and published. While the data collection methods naturally vary among Hofstede's, Schwartz's, Lynn and Vanhanen's studies and in EPQ data, it should be noted that all these studies provide comparable country scores, which can be used in cross-cultural comparisons. Similarly, CPI and GDP are measured annually by using the same methodology, which means that the country scores are comparable.

The data were analyzed by means of Pearson product-moment and partial correlations and hierarchical regression analyses, which were also used to test mediation and moderation models. The models were analyzed separately because the aim was to compare different cultural models to each other. Since the models are partly overlapping, leading to inevitable multicollinearity problems and because of the limitations related to the sample size (number of counties), separate analyses were the only methodological option.

## 3 Results

### 3.1 Correlations and partial correlations between CPI and Hofstede's measures, Schwartz's value scores, EPQ and IQ

In order to understand direct relationships between the predictor variables (independent variables) and the corruption score (dependent variable), pairwise Pearson product-moment correlations were calculated. Since the predictor variables are likely to have correlations with the GDP and each other, also partial correlations were calculated. In the first partial correlations, only GDP was controlled, i.e., the effect of GDP partialed out. In the second partial correlation, all the other variables in the model were controlled, i.e. their effects partialed out. Comparison of these correlations shows how much the individual cultural characteristics account variance for in corruption scores when the effects of income (GDP) level and all other variables have been cleaned off.

Pearson correlation coefficients (based on two-tailed probability testing) and partial correlation coefficients between CPI scores and study variables are presented in Table 2. Table 2 shows that EPQ Lie score, PDI, UAI, IDV, Conservatism, intellectual autonomy, hierarchy, egalitarianism, and IQ correlated statistically significantly with CPI. However, when GDP per capita was controlled, only MAS and IQ had a significant relationship to CPI. When all the remaining variables were controlled, the MAS and mastery had statistically significant relationships with the CPI score. Hence, the national income seems to be an essential factor, which influences (suppresses) the relationships between study variables and the CPI.

**Table 2:** Correlations between CPI score and study variables: Pearson correlation coefficients, partial correlation coefficients in which GDP per capita was controlled and partial correlation coefficients in which all other variables were controlled.

	Correlation	Partial correlation (only GDP controlled)	Partial correlation (all other variables controlled)
EPQ			
Psychoticism	0.198	0.133	-0.035
Extraversion	-0.144	-0.086	0.137
Neuroticism	0.182	0.189	0.087
Lie	0.654∗∗	0.149	0.113
Hofstede's measures			
PDI	0.654∗∗	0.230	0.185
UAI	0.349∗	0.209	0.202
IDV	-0.695∗∗	-0.088	-0.089
MAS	0.189	0.327∗	0.323∗
Schwartz’ values			
Conservatism	0.456∗	-0.352	0.218
Affective autonomy	-0.350	0.162	0.018
Intellectual autonomy	-0.367∗	0.368	0.308
Hierarchy	0.398∗	0.032	0.256
Mastery	0.359	0.374	0.509∗
Egalitarianism	-0.416∗	0.088	0.164
Harmony	0.035	0.262	0.356
Intelligence (IQ)	-0.632∗∗	-0.254∗	-0.251

Note: sample sizes for EPQ, Hofstede, Schwartz, and IQ were 35, 46, 30, and 64, respectively. GDP = gross domestic product per capita.

∗ p < 0.05.

∗∗ p < 0.01.

### 3.2 Regression analyses for predicting the CPI score

Comparison between correlations, partial correlations controlling the GDP, and partial correlation controlling all the other variables indicated that GDP and the other variables have an effect on the relationship between a cultural variable (Hofstede, Schwartz, EPQ, IQ) and corruption. To further clarify these relationships, two sets of regression analyses were calculated: the first without GDP and the second with GDP entered the model (controlled) in the first step. The first set of regression analyses shows how cultural models account for the variance in corruption. The second set of regression analyses shows the effect of cultural variables after the effects of GDP have been controlled. Both analyses are essential for the purpose of the study because the first type of analysis allows us to compare the predictive power of cultural models in corruption, while the second set of analyses allow us to see how the role of GDP per capita in each model. From the theoretical point of view, both analyses are justifiable depending on the point of interest.

Four regression analyses were conducted (CPI score as the dependent variable) to investigate how Hofstede measures Schwartz values, EPQ, and IQ predict CPI scores. These four models were tested separately because the aim was to compare different models and because lacking data prevented the inclusion of all variables into the same model. Results of the regression analyses without GDP per capita and separately for EPQ, Hofstede, Schwartz, and IQ models are listed in Table 3.

**Table 3:** Multiple regression analysis results predicting CFI score.

Independent variables	B			Std. Error		β	t	
EPQ (R^2^ = 0.485; F_4,28_ = 6.590∗∗∗)								
Psychoticism	0.293			0.291		0.150	1.004	
Extraversion	0.088			0.152		0.084	0.575	
Neuroticism	0.144			0.147		0.146	0.984	
Lie	0.558			0.115		0.690	4.852∗∗∗	
Hofstede's measures (R^2^ = 0.599; F_4,48_ = 16.418∗∗∗)								
Power Distance	0.033			0.015		0.295	2.245∗	
Uncertainty avoidance	0.013			0.010		0.130	1.278	
Individualism	-0.045			0.013		-0.464	-3.428∗∗∗	
Masculinity	0.026			0.013		0.201	2.073∗	
Schwartz’ values (R^2^ = 0.602; F_7,22_ = 4.756∗∗)								
Conservatism	6.737			3.288		0.978	2.049	
Affective autonomy	0.873			1.329		0.158	0.656	
Intellectual autonomy	1.243			1.688		0.222	0.736	
Hierarchy	2.118			1.015		0.458	2.087∗	
Mastery	5.140			1.637		0.565	3.139∗∗	
Egalitarianism	1.677			2.056		0.274	0.816	
Harmony	3.123			0.943		0.616	3.313∗∗	
IQ (R^2^ = 0.399; F_1,61_ = 40.535∗∗)								
Intelligence (IQ)		-0.165	0.026		-0.632			-6.367∗∗∗

Note: sample sizes for EPQ, Hofstede, Schwartz, and IQ were 35, 46, 30, and 64, respectively.

∗ p < 0.05.

∗∗ p < 0.01.

∗∗∗ p < 0.001.

Findings of the regression analyses showed that the EPQ Lie scale was the best and only significant predictor (49% of the variance in CPI score accounted for) among EPQ variables. The high EPQ Lie score was related to a high level of corruption (beta = 0.69). Among Hofstede's measures, PDI, IDV, and MAS predicted CPI scores significantly. The total amount of variance accounted for was 60%, and the significant predictors were IDV, PDI, and MAS. A high level of individualism was negatively related to corruption, whereas high power distance and masculinity were positively related to CPI scores. Schwartz's values predicted the same amount of variance as Hofstede's measures, i.e., 60%. Hierarchy, mastery, and harmony were all positively related to high CPI scores. Interestingly, IQ alone predicted 40% of the variance in CPI score: countries scoring high in IQ scored lower in CPI than countries with low IQ.

Table 2 already showed that GDP per capita had a substantial effect on the relationships between CPI and predictor variables. In the next set of regression analyses, similar regression analyses, as described in Table 3, were conducted except that GDP per capita was entered into the model in the first step. Hence, the independent contribution of study variables after controlling GDP per capita was investigated. Table 4 shows that the predictive power of the other variables dropped drastically when GDP per capita was forced into the model in the first step. In the EPQ model, none of the variables was significantly related to the CPI score. Among Hofstede's measures, only MAS was related to the CPI score after forcing GDP to the model. Similarly, only Schwartz's mastery had an independent contribution to the model after forcing GDP per capita to the model. As Table 4 shows, IQ accounted for 2% of the variance. GDP is obviously a very strong predictor of corruption. In order to further investigate the role of GDP in culture – corruption relationship, mediator and moderator analyses were conducted.

**Table 4:** Multiple regression analysis results predicting CFI score after controlling for GDP per capita.

Independent variables	B	Std. Error	β	t
EPQ (Model 1: R^2^ = 0.810; F_1,31_ = 132.390∗∗∗; Model 2: R^2^ = 0.816; F_5,27_ = 24.003∗∗∗)				
Model 1				
GDP	0.000	0.000	-0.900	-11.506∗∗∗
Model 2				
GDP	0.000	0.000	-0.862	-6.980∗∗∗
Psychoticism	-0.034	0.183	-0.017	-0.183
Extraversion	0.066	0.093	0.063	0.717
Neuroticism	0.041	0.090	0.041	0.451
Lie	0.059	0.100	0.073	0.588
Hofstede's measures (Model 1: R^2^ = 0.735; F_1,46_ = 127.734∗∗∗; Model 2: R^2^ = 0.786; F_5,42_ = 30.929∗∗∗)				
Model 1				
GDP	0.000	0.000	-0.857	-11.302∗∗∗
Model 2				
GDP	0.000	0.000	-0.687	-6.004∗∗∗
PDI	0.014	0.012	0.125	1.216
UAI	0.010	0.008	0.101	1.335
IDV	-0.007	0.012	-0.71	-0.579
MAS	0.021	0.010	0.161	2.214∗
Schwartz’ values (Model 1: R^2^ = 0.705; F_1,27_ = 64.523∗∗∗; Model 2: R^2^ = 0.815; F_8,20_ = 11.000∗∗∗)				
Model 1				
GDP	0.000	0.000	-0.840	-8.033∗∗∗
Model 2				
GDP	0.000	0.000	-0.745	-4.604∗∗∗
Conservatism	2.521	2.530	0.358	0.997
Affective autonomy	0.078	0.970	0.014	0.081
Intellectual autonomy	1.766	1.222	0.309	1.446
Hierarchy	0.927	0.783	0.198	1.184
Mastery	3.265	1.236	0.359	2.642∗
Egalitarianism	1.096	1.475	0.177	0.743
Harmony	1.369	0.804	0.270	1.704
IQ (Model 1: R^2^ = 0.766; F_1,60_ = 196.695∗∗∗; Model 2: R^2^ = 0.781; F_2,59_ = 105.183∗∗∗)				
Model 1				
GDP	0.000	0.000	-0.875	-14.025∗∗∗
Model 2				
GDP	0.000	0.000	-0.779	-10.017∗∗∗
Intelligence (IQ)	-0.041	0.021	-0.155	-1.990

Note: sample sizes for EPQ, Hofstede, Schwartz, and IQ were 35, 46, 30, and 64, respectively.

GDP = gross domestic product per capita.

∗p < 0.05; ∗∗p <0 .01; ∗∗∗p < 0.001.

### 3.3 Mediator and moderator analyses

Regression analysis results listed in Tables 2 and 3 clearly show that national income (GDP per capita) is an important variable influencing the relationships between the study variables and the CPI score. Without taking into account the role of GDP, the relationships between culture, values, national personality, and IQ cannot be fully explained.

**Figure 1:**
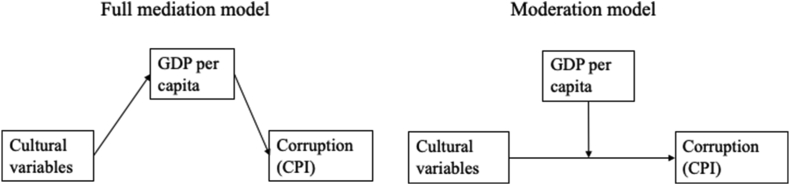
Theoretical models tested in mediation and moderation analyses.

Earlier empirical studies show that many cultural variables have relationships to GDP and that GDP is a strong predictor of corruption level in a country. From a theoretical point of view, however, the GDP per capita can either mediate or moderate the influence of cultural variables on corruption. These two theoretical models about mediator-moderator roles of GDP are described in Figure 1. The first model describes a full mediator model in which the effect of a cultural variable/personality characteristic/IQ on corruption (CPI) is mediated by national income. This would mean that the culture either promotes or prohibits economic prosperity, which in turn influences the corruption level in a county. In the case of moderation, the culture – corruption relationship (positive, negative or neutral) would depend on the national income level.

A moderator effect exists when a variable influences the direction or the strength of the relationship between an independent variable (IV) and a dependent variable (DV) (Baron and Kenny, 1986). In this study, regression analysis was used to investigate the moderation effects of GDP per capita. Before applying the regression analyses, the IV in concern and the moderator (GDP per capita) were standardized, and a moderator term (IV∗GDP per capita) was calculated. Variables for moderator and mediator analyses were chosen based on the results listed in Table 3: only those variables were selected, which had a significant independent contribution to CPI score in regression analyses. Consequently, seven regression analyses for moderation were conducted.

In the mediation model, the IV (predictor) affects a second variable (mediator), which, in turn, affects the DV (outcome). To study whether the effects of Hofstede's measures, Schwartz's values, EPQ, and IQ on CPI were mediated by GDP per capita, the procedure suggested by (Baron and Kenny, 1986) was applied.

#### 3.3.1 Moderation and mediation effects of GDP per capita on Hofstede's variables -CPI relationship

Table 3 shows that statistically significant relationships between PDI, IDV, and MAS, and CPI were found. Hence, three separate moderator and mediator analyses were run.

Moderator analyses indicated that only the IDV – CPI relationship was moderated by GDP per capita (beta coefficient for the moderator = 0.220; t = 2.412, p < 0.05). No moderation effect was found in MAS and PDI.

**Figure 2:**
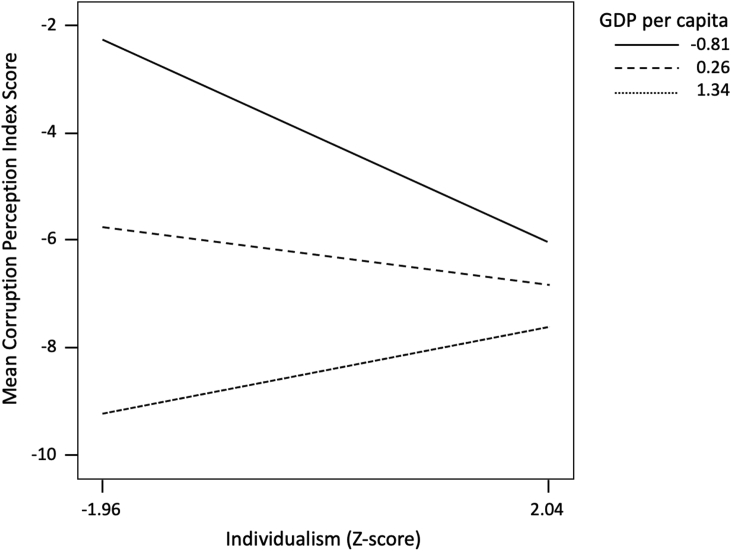
The interaction between Hofstede's individualism score (z-score) and GDP per capita (z-score) on the Corruption Perception Index score.

The SIMPLE syntax program (O'Connor, 1998) was used to display statistically significant interactions, which were plotted by calculating simple regression equations of the DV at low (−1 SD below M), moderate (M), and high (+1 SD above M) levels of GDP per capita (Aiken and West, 1991). Figure 2 shows the interaction between IDV and GDP per capita on CPI scores. Among the low-income countries, increasing IDV was related to the reduction in the CPI scores, while among the high-income countries, an increase in IDV was positively related to CPI scores. However, only the slope of the low-income countries was statistically significant (beta = -0.374; t = -2.455, p < 0.05).

Among Hofstede's variables, mediation effects of GDP per capita were found for PDI and IDV but not for MAS. The effect of PDI on CPI was fully mediated. In the case of PDI, the absolute value of the beta coefficient of the IV in the 3^rd^ equation decreased from 0.654 (p < 0.001) to 0.169 (p = NS), indicating full mediation. Similarly, the beta coefficient for IDV changed from –0.695 (p < 0.001) to –0.121 (p = NS), indicating full mediation.

#### 3.3.2 Moderation and mediation effects of GDP per capita on Schwartz's values -CPI relationship

Three value scales – hierarchy, mastery, and harmony – predicted CPI scores significantly (Table 3). These variables were chosen for moderator and mediator analyses. Moderator analyses showed no moderation effect for any of the Schwartz value types.

The mediator effect of GDP per capita was found only for “Hierarchy”: The value of the beta coefficient of the independent variable in the 3^rd^ equation decreased from 0.398 (p < 0.05) to 0.036 (p = NS), indicating full mediation.

#### 3.3.3 Moderation and mediation effects of GDP per capita on EPQ-CPI relationship

Table 3 shows that only the EPQ Lie scale had a significant relationship with the CPI score. Therefore, only EPQ Lie – CPI moderation and mediation models were tested. In the moderation analysis, no moderation effects were found. In mediation analysis, all the required conditions were met, and full mediation from EPQ Lie via GDP per capita to CPI was found. The beta coefficient for EPQ Lie dropped from 0.654 (p < 0.001) to 0.051 (p = NS). Hence, the EPQ Lie scale had no direct effect on CPI, but its effect was fully mediated by GDP per capita.

#### 3.3.4 Moderation and mediation effects of GDP per capita IQ -CPI relationship

Table 3 shows a strong relationship between IQ and the CPI score. No moderation effect was found in moderation analysis. However, mediation analysis showed that the GDP per capita mediated the effects of IQ on CPI: the beta coefficient for IQ changed from –0.632 (p < 0.001) to -0.155 (p = 0.051).

## 4 Discussion

### 4.1 Hofstede's cultural dimensions and corruption

The findings of the association between Hofstede's cultural dimensions and corruption have been somewhat mixed. In most of the studies, UAI and PDI have been positively related to corruption (Cheung and Chan, 2008; Connelly and Ones, 2008; Davis and Ruhe, 2003; Franke and Nadler, 2008; G. Hofstede, 2001; Husted, 1999; Yeganeh, 2014). The strong PDI – corruption relationship is easy to understand because, in high PDI countries, it is accepted that different rules apply to people in higher positions than to people in lower positions. Consequently, the services provided by state bureaucrats to citizens are not seen as “rights of citizens” but as “favours” that depend on the goodwill of the bureaucrat. Consequently, people with lower social status are entitled to give bribes or “presents” to the officers belonging to the higher status to speed up or facilitate the process related to their request. The relationship between UAI and corruption is also simple. If citizens feel that they are incompetent and helpless toward authorities and should leave decision making to “experts”, the authorities have greater temptations to accept bribes, and the citizens have fewer opportunities to control the authorities (Hofstede, 2001, p. 173). Also, MAS has been reported to be positively related to corruption in some studies (Davis and Ruhe, 2003; Hofstede, 2001; Tu et al., 2020; Yeganeh, 2014), while this relationship has not been found in several studies (Cheung and Chan, 2008; Connelly and Ones, 2008; Franke and Nadler, 2008). Thus, the results about MAS and corruption have been inconclusive, although we could suppose that in masculine competition-driven society, corruption is more commonly accepted as means for success than in societies emphasizing collaboration. IDV seems to be negatively related to corruption (Connelly and Ones, 2008; Davis and Ruhe, 2003; Tu et al., 2020; Yeganeh, 2014), but that relationship is highly dependent on GDP per capita (Hofstede, 2001; Husted, 1999).

In the present study, high PDI and MAS scores and low IDV scores were significant predictors of corruption when GDP per capita was not entered in the model. After the inclusion of GDP per capita, only MAS predicted corruption scores. Moderation and mediation analyses were run to study further the role of national income on culture – corruption –relationship. Moderation analyses showed that the effect of IDV on corruption score was moderated by GDP per capita so that in low-income countries, IDV was negatively and in high-income countries positively related to corruption. The combination of collectivism and poverty may make a country vulnerable to corruption. One reason for this finding might be that in developing countries, the quality of governance is often low, and control of corruption is lacking, which makes corruption easy and the likelihood of being caught and punished low. Institutionalized bribery and in-group favouritism might even replace the rules, and the usual administrative practices and the network of relatives and friends may partly take the role of state institutions. The mediation analyses showed that the effects of IDV and PDI on corruption were fully mediated by GDP per capita, i.e., IDV and PDI were related to the income of a nation, and the income, in turn, was related to corruption. This result suggests that changing cultural values of power distance and individualism might not be an effective strategy for reducing corruption because a change in these variables should first improve the economic situation in a country, which, in turn, would lead to a decrease in corruption.

### 4.2 Schwartz's cultural values and corruption

Previous studies about Schwartz's values and corruption have focused on different aspects or used different indexes than those of the present study, which makes results somewhat incomparable. Yeganeh (2014) studied Schwartz's values among 55 countries and found that Conservatism and harmony were positively related to corruption, while autonomy as well as mastery were negatively related to corruption. On the other hand, Mazzi et al. (2018) reported hierarchy and mastery to be negatively related to mandatory accounting disclosures in European companies. In company data from five countries, Agyei-Mensah and Buertey (2019) reported a negative correlation between mastery and corruption, while embeddedness correlates positively with corruption.

In this study, Schwartz's cultural values hierarchy, mastery, and harmony predicted corruption when GDP per capita was not controlled, whereas after controlling GDP per capita, only mastery was related to corruption. Mastery refers to values in which priority is in the dominance of surroundings through self-assertion. Nations scoring high in mastery also score high in corruption because the essence of mastery is in dominance by using any means – even illegal ones. Mediator analysis showed full mediation of GDP per capita between hierarchy and corruption: nations emphasizing high hierarchy seemed to be less wealthy, which, in turn, was related to a higher amount of corruption. The relationship between hierarchy, income, and corruption is as expected because acceptance of stability of the hierarchical roles is related to uneven resource allocation, which, in turn, is closely related to corruption.

### 4.3 Individual difference factors and corruption

Results of the present study are in line with those of van Hemert et al. (van Hemert et al., 2002): national EPQ Lie scale score was positively related to corruption. Psychoticism, however, was not statistically significantly associated with corruption. One reason for the statistically non-significant correlation between EPQ P and CPI might be that psychoticism is the least reliable of the EPQ scales. The other possibility is that since psychoticism is a personality trait characterized by antisocial attitudes and norm violations, it does not promote in-group loyalty and social exchange necessary for “successful” corruption (e.g., favouritism). People scoring high in psychoticism simply do not “fit in” because of their antisocial tendencies and, therefore, are left out from social networks. It is important to bear in mind that psychoticism does not refer to “psychosis” or to mental disorders such as schizophrenia or schizotypal personality disorder, but “psychoticism” is closer to toughminded, unconsidered, and reckless personality characteristics, which might lead to social isolation and fewer opportunities for establishing collaboration and networks of mutual favours.

According to the present study, EPQ L – corruption –the relationship was fully mediated by national income. Nations scoring high in the Lie scale tend to be poorer and, thus, more corrupt. The EPQ Lie scale - despite its name – does not actually measure lying as dishonesty when answering questionnaires but as a personality trait in which a good impression is seen as more important than genuine presentation of self (Furnham et al., 2008). Societies scoring high on the Lye scale emphasize appropriate behaviour adjusted to the role in the family or in society. Expression of negative characteristics or weaknesses is not encouraged. Bribes as “presents” might be used as a means of giving a good impression and favours to other people; such as nepotism can be seen as appropriate or natural behaviour within the family or the community. In this sense, corruption might be just a way to express one's commitment and loyalty to the community. Connelly and Ones (2008) reported a relationship between neuroticism, extraversion, and corruption. These relationships were not found in the present study. One of the reasons for different results between the present study and van Hemert's study and Connelly's and Ones' (2008) study might be that Connelly and Ones used NEO-PI for measuring personality and, thus, did not have EPQ Lie score available.

Lynn and Vanhanen (Lynn and Vanhanen, 2002, 2006) reported a correlation of -0.59 between IQ and corruption. Similarly, some other studies have reported that high cognitive abilities (IQ) predict lower corruption perceptions (Achim et al., 2021; Farzanegan, 2020; Potrafke, 2012; Salahodjaev and Malikova, 2021). According to the findings of the present study, IQ has a strong negative correlation (r = -0.63) to corruption when GDP per capita was not controlled. This correlation remained statistically significant, although it decreased when GDP was controlled. The mediation analysis showed that GDP per capita had a mediator role between IQ and corruption. Obviously, establishing well working social and governmental institutions required for control of corruption and efficient management requires human capital and, thus, intelligence. Another explanation is that long-term time horizons require intelligence instead of fulfilling immediate needs (Potrafke, 2012). Since intelligence measured by intelligence tests is a very abstract concept, it is actually difficult to say how exactly intelligence is related to corruption except as a predictor of high GDP per capita. The relationship between tertiary education attendance rates and corruption was reported earlier (Cheung and Chan, 2008).

### 4.4 Limitations of the study

This study has some limitations, which should be considered when evaluating the significance of the results. Firstly, the study is based on correlational analyses of country-level data representing public sector corruption which is undoubtfully a rough approach ignoring the delicate mechanisms of how corruption works in different societies and different sectors (e.g., private sector). On the other hand, as the present study shows, macro-level analyses can reveal some crude mechanisms of how culture and culturally dominant individual difference factors can be related to national income and corruption.

Secondly, different cultural models (Hofstede, Schwartz, EPQ, IQ) were tested separately, i.e., the set of countries analyzed was somewhat different in each case. The reason for this methodological choice is pragmatic: requiring a full dataset including values for the same countries for all variables would result in a very small set of countries, which would not be enough for any kind of analysis. Nevertheless, it should be noted that separate analyses can restrict the generalizability of the results even if the core sets of the countries are very similar in terms of the key cultural and economic variables. None of the models was predominantly more “Western” or “rich” than the others. Still, it should be borne in mind that the datasets were not equal, and some variation in results can be attributed to the samples.

Thirdly, while being used widely in research, the variables included in the current study have been subjected to criticism. For example, some researchers (Bello y Villarino, 2021; Lin and Yu, 2014) have criticized the Corruption Perceptions Index (CPI) for ignoring the local perspectives, which might bias the estimates and undermine the comparability among countries. Despite its limitations, CPI provides a standardized tool for measuring crude corruption in different countries and, thus, allows international comparisons at the general level. Moreover, GDP per capita suffers from the same cultural insensitivity as CPI. As Morse (2008) pointed out, the assumption of GDP per capita as an indicator of ‘successes and ‘achievement’ is a highly reductionist way to measure cultures because it is deeply rooted in Western values. In the present study, we did not attempt to quantify a country's success with GDP but rather assumed that nations' income levels may determine the existing economic realities and, thus, the role of corruption in society.

Fourth, national-level measurements of cultural values and individual differences (such as IQ and personality) suffer from the shortcomings mentioned above and ignore the details of local cultures. The general assumption is that cultures and national personality characteristics can be summarized with a few key factors (e.g., Schwartz's and Hofstede's value dimensions, individual difference factors) and that these characteristics change slowly. In addition, the third assumption underlying these measures is that such concepts as intelligence and personality can be measured with the same validity in different cultures. The adequacy of these assumptions has been questioned in some studies (Morse, 2008; Richardson and Norgate, 2014). While these criticisms about culture dependency and reduced validity in certain cultures have to be taken with great concern, all macro-level measures of cultural characteristics suffer from the same tendency to reductionism and cultural insensitivity. If we believe that macro-level comparisons among countries can reflect some important mechanisms of how culture interacts with the economic and political reality (e.g., level of corruption, income), we have to rely on these indicators while acknowledging all the shortcomings they have. It should also be noted that these analyses aim to describe macro-level mechanisms and not to rank cultures to ‘successful’ and ‘unsuccessful’ ones.

## 5 Conclusions

The results of this study emphasized the importance of cultural values, personality, and IQ as antecedents of corruption. National EPQ Lie scale scores, PDI, IDV, MAS, Hierarchy, Mastery, Harmony and especially intelligence predicted the national CPI scores. When GDP per capita was controlled, only MAS and Mastery predicted CPI scores. Mediation analyses showed, however, that the effects of several cultural factors (IDV, PDI, MAS, Hierarchy, EPQ Lie) on corruption were mediated by GDP. Hence, these factors first influence the national GDP per capita, and then, in turn, GDP influences corruption. Moderation analyses indicated that only the IDV – CPI relationship was moderated by GDP per capita. The dominant role of income does not undermine the importance of cultural factors but suggests that cultural factors are distal factors influencing corruption via proximal factors like economic situation. Since political and economic decisions are based on cultural values and preferences and not vice versa, it can be expected that cultural values function as background factors behind decisions related to anti-corruption policies. These results suggest that cultural factors should be considered in corruption studies and anti-corruption policies. Without considering the national cultural values, anti-corruption policies might not be perceived well by the public, compromising the effects of the interventions. Besides, the interaction between the national income and cultural values should be taken into account, because the influence of cultural values on corruption can depend on the income level of the country. In sum, this study highlights the complex mechanisms how national culture and income are related to the likelihood of corruption.

## Declarations

### Author contribution statement

Esma Gaygısız; Timo Lajunen: Conceived and designed the experiments; Performed the experiments; Analyzed and interpreted the data; Contributed reagents, materials, analysis tools or data; Wrote the paper.

### Funding statement

This research did not receive any specific grant from funding agencies in the public, commercial, or not-for-profit sectors.

### Data availability statement

Data will be made available on request.

### Declaration of interests statement

The authors declare no conflict of interest.

### Additional information

No additional information is available for this paper.
