# Editorial note to: “Cultural values, national personality characteristics, and intelligence as correlates of corruption: A nation level analysis” [Heliyon 8(5) (August 2022) e09506]

**DOI:** 10.1016/j.heliyon.2023.e23820

**Published:** 2023-12-20

**Authors:** Esma Gaygısız, Timo Lajunen

**Affiliations:** a Department of Economics, Middle East Technical University, 06531, Ankara, Turkey; b Department of Psychology, Norwegian University of Science and Technology, 7491, Trondheim, Norway

Heliyon is publishing an Editorial Note regarding the following article “Cultural values, national personality characteristics, and intelligence as correlates of corruption: A nation level analysis” by Gaygısız and Lajunen. We were informed by a reader of this paper that the use of the datasets presented in “IQ and the Wealth of Nations” by Lynn, R. and Vanhanen, T., 2002, and “IQ and global inequality”, Lynn, R. and Vanhanen, T., 2006, and their inclusion in the analysis of IQ/intelligence as an antecedent of corruption, is highly disputed and no longer considered valid to include in the article's findings.

The other three analyses in the article related to cultural values and personality characteristics and their relationship to corruption were each run separately, and therefore their findings remain unchanged by the exclusion of the above two datasets. The authors have agreed to the publication of this Editorial Note.

